# Evaluation of bio-efficacy of field-aged novel long-lasting insecticidal nets (PBO, chlorfenapyr or pyriproxyfen combined with pyrethroid) against *Anopheles gambiae* (*s.s.*) in Tanzania

**DOI:** 10.1016/j.crpvbd.2024.100216

**Published:** 2024-09-23

**Authors:** Jackline L. Martin, Louisa A. Messenger, Edmund Bernard, Monica Kisamo, Patric Hape, Osca Sizya, Emmanuel Festo, Wambura Matiku, Victoria Marcel, Elizabeth Malya, Tatu Aziz, Nancy S. Matowo, Jacklin F. Mosha, Franklin W. Mosha, Mark Rowland, Alphaxard Manjurano, Natacha Protopopoff

**Affiliations:** a Department of Parasitology, Pan-African Malaria Vector Research Consortium, Kilimanjaro Christian Medical University College, BOX 2240, Moshi, Tanzania; b Department of Parasitology, National Institute for Medical Research, BOX 1462, Mwanza, Tanzania; c Department of Disease Control, Faculty of Tropical Diseases, London School of Hygiene and Tropical Medicine, London, WC1E 7HT, UK; d Department of Environmental and Occupational Health, School of Public Health, University of Nevada, Las Vegas, NV, 89119, USA; e Parasitology and Vector Biology (PARAVEC) Laboratory, School of Public Health, University of Nevada, Las Vegas, NV, 89119, USA; f Health Interventions Unit, Department of Epidemiology and Public Health, Swiss Tropical & Public Health Institute, 4123, Allschwill, Switzerland

**Keywords:** Long-lasting insecticidal nets, Dual-active ingredient insecticide-treated nets, Insecticide resistance, Malaria vectors, Bio-efficacy, Cone assays, Tunnel test, Tanzania

## Abstract

Next-generation insecticide-treated bed nets (ITNs) combining two insecticides or an insecticide with a synergist are vital in combating malaria, especially in areas with pyrethroid-resistant mosquitoes where standard pyrethroid long-lasting insecticidal net (LLIN) may be less effective. A community durability study was conducted in Misungwi, Tanzania, during a cluster randomised controlled trial. This study assessed the bio-efficacy of three net brands combining a pyrethroid insecticide and either a synergist PBO for Olyset Plus, or a second insecticide pyriproxyfen for Royal Guard, and chlorfenapyr for Interceptor G2 over three years. These nets were compared to Interceptor, a standard pyrethroid-only net. A total of 1950 nets were enrolled across 10 clusters in each treatment arm. Thirty nets per type were collected every 6 months up to 30 months, with 50 nets sampled at 36 months. WHO cone bioassays and tunnel tests were performed at 0, 12, 24, 30 and 36 months. Both susceptible *An. gambiae* (*s.s.*) Kisumu strain and resistant *An. gambiae* (*s.s.*) Muleba-Kis strain were exposed. Over 80% of nets tested against the susceptible Kisumu strain met the WHO criteria after three years of community use. In tunnel tests, mortality (72 h) of the resistant *Anopheles* varied between 52% and 20%, in Interceptor G2 and was higher than standard Interceptor net up to 24 months. Olyset Plus mortality (24 h) ranged between 84% and 33% in tunnel tests with superior efficacy compared to Interceptor at 0, 24 and 36 months. Sterility effects in Royal Guard were higher when these nets were new and at six months but decreased to less than 10% after 12 months. Royal Guard consistently induced higher mortality compared to Interceptor up to 30 months while next-generation ITNs demonstrated higher efficacy in terms of mortality compared to standard LLINs against resistant strains; this superior bio-efficacy did not persist for the full three years. The impact of active ingredient (dual-AI) and PBO diminished relatively quickly. Aside from the initial period when the nets were new, the differences in mortality for Interceptor G2 and Olyset Plus and in sterility for Royal Guard, compared to the standard LLINs, were relatively small thereafter.

## Highlights

• The bio-efficacy of Interceptor G2, Royal Guard, and Olyset Plus was tested against a standard LLIN over three years.
• Nets were evaluated under community use conditions to compare long-term effectiveness in real settings.
• All nets met the WHO bio-efficacy criteria in cone and tunnel tests against a susceptible mosquito strain.
• Next-generation nets showed superior killing effects compared to standard LLINs against resistant mosquito strains.
• Overall, next-generation nets performed better over time in controlling resistant vectors than standard pyrethroid LLINs.

## Graphical abstract

**Figure:**
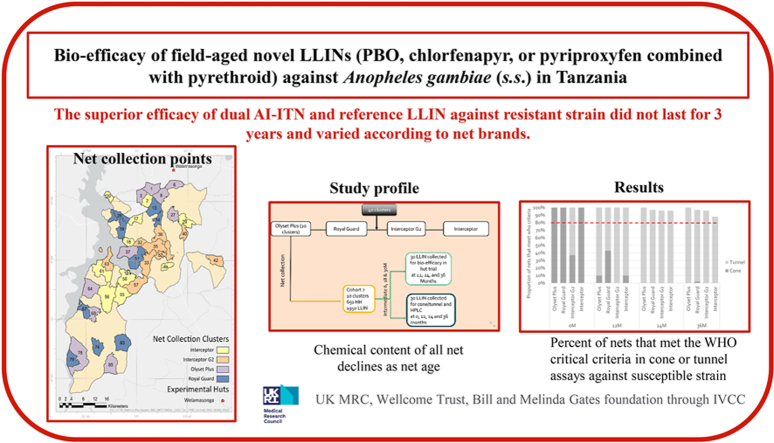

## 1 Introduction

Vector control interventions have played a crucial role in reducing malaria, averting an estimated 2.1 billion cases (82%) and 11.7 million deaths (94%) in sub-Saharan Africa between 2000 and 2022, with long-lasting insecticidal nets (LLINs) being a major contributor (WHO, 2023a). Standard LLIN has faced different challenges including widespread insecticide resistance which may have contributed to the increase in malaria cases globally since 2015. In 2022, there were 5 million more cases reported in the sub-Saharan region, compared to 2021 (WHO, 2023a).

In areas where mosquitoes have developed resistance to pyrethroids, user protection is diminished because fewer mosquitoes are killed after exposure to the net, allowing them to survive long enough to transmit the parasite. In addition, the barrier offered by the pyrethroid is reduced, enabling mosquitoes to penetrate through holes in the nets and feed on human hosts. Both the reduction in killing and the increase in blood-feeding may impact malaria transmission (Martin et al., 2021). To address this challenge, various insecticide nets (ITNs) treated with two insecticides or with a pyrethroid insecticide and a synergist have been recommended by the World Health Organisation (WHO, 2023a) as they show superior efficacy on malaria outcomes compared to standard LLINs (Protopopoff et al., 2018; Mechan et al., 2022; Mosha et al., 2022, 2023; Accrombessi et al., 2023). In the context of WHO prequalification, insecticide-treated bed nets (ITNs) are defined as mosquito nets treated with active ingredients (AIs) aimed at repelling or killing *Anopheles*, thereby providing both personal and community protection (WHO, 2023b).

Olyset Plus, a net combining PBO and the pyrethroid permethrin, outperformed standard Olyset net against multi-resistant wild *Anopheles* mosquitoes in experimental huts (Pennetier et al., 2013). Similar results were reported in Cameroon after using the first generation (F1) from the crossing between a highly resistant *Anopheles funestus* (FUMOZ) and a susceptible *An. funestus* colony (FANG) (Menze et al., 2022) and in Burkina Faso with significant mortality in resistant malaria vector species compared to standard pyrethroid nets (Toe et al., 2018; Menze et al., 2022) in experimental hut trials (EHTs).

Royal Guard (containing the pyrethroid alpha-cypermethrin, and the juvenile hormone growth inhibitor pyriproxyfen) met the WHO criteria, achieving 95% knockdown and more than 80% mortality for up to 20 washes against resistant *An. gambiae* (*s.s.*) (Kisumu strain) in cone assays (Ngufor et al., 2020). It also met the WHO criteria in tunnel tests, with mortality exceeding 80% after 20 washes using the same strain. This net was also more effective against wild pyrethroid-resistant vectors (increase in sterility and mortality) in EHT (Ngufor et al., 2020) in Benin.

Interceptor G2 (containing alpha-cypermethrin and the pyrrole chlorfenapyr) was able to induce 71% mortality against resistant, free-flying *An. gambiae* (*s.l.*) in an EHT, compared to an alpha-cypermethrin-only net (20% mortality) (NʼGuessan et al., 2016). When Interceptor G2 and Royal Guard were assessed in other studies using washed nets, the performance of dual-AI ITNs remained superior even after 20 washes compared to reference nets in EHT and laboratory settings (NʼGuessan et al., 2016; Ngufor et al., 2020; Tungu et al., 2021).

In order to assess the effective life span of the net under routine use, insecticide bio-efficacy and physical durability of naturally aged nets need to be monitored in a community study setting for a minimum of three years (Ngufor et al., 2020; WHO, 2023b). Previous studies reported that standard pyrethroid-only nets have demonstrated 3-year long lasting efficacy; however, there is less evidence of the long-lasting bio-efficacy of the second partner insecticide (pyriproxyfen/chlorfenapyr) or PBO when these nets age. Bio-efficacy is measured by the ability of ITNs to induce mosquito mortality, knockdown, prevent blood-feeding or sterility for some insecticide in laboratory bioassays using susceptible and resistant mosquito strains (WHOPE, 2013).

This study aims to assess the bio-efficacy against entomological outcomes of dual-AI ITNs and PBO-pyrethroid ITNs compared to standard pyrethroid LLINs collected from the community over three years.

## 2 Materials and methods

### 2.1 Study design

The laboratory cone and tunnel assays were performed using naturally aged nets sampled from Misungwi community and new unwashed nets against the susceptible Kisumu strain and the resistant Muleba-Kis strain of *An. gambiae* (*s.s*.) (Martin et al., 2022).

The following ITNs were assessed: (i) Royal Guard (Disease Control Technologies, LLC, Greer, SC, USA), a polyethylene net combining 225 mg/m^2^ pyriproxyfen, which is known to disrupt female mosquito reproduction and egg fertility, and 261 mg/m^2^ of pyrethroid alpha-cypermethrin; (ii) Interceptor G2 (BASF Corporation, Ludwigshafen, Germany), ITN made of polyester incorporating two adulticides with different modes of action, 200 mg/m^2^ chlorfenapyr and 100 mg/m^2^ alpha-cypermethrin. The chlorfenapyr disrupts the insectʼs ability to convert energy. (iii) Olyset Plus (Sumitomo Chemicals, Tokyo, Japan) is a polyethylene ITN which incorporates a synergist, 400 mg/m^2^ piperonyl butoxide (PBO), to enhance the potency of the partner pyrethroid insecticide and 800 mg/m^2^ permethrin; (iv) Interceptor (BASF Corporation, Germany) net (positive control net), which contains alpha-cypermethrin at a target dose of 200 mg/m^2^ on polyester fabric. Untreated nets were purchased from the local market and used as a negative control.

Fabric integrity and survivorship of the ITNs was assessed in this study through longitudinal surveys and was reported elsewhere (Martin et al., 2024).

### 2.2 Sample size and sampling of ITNs

**Fig. 1:**
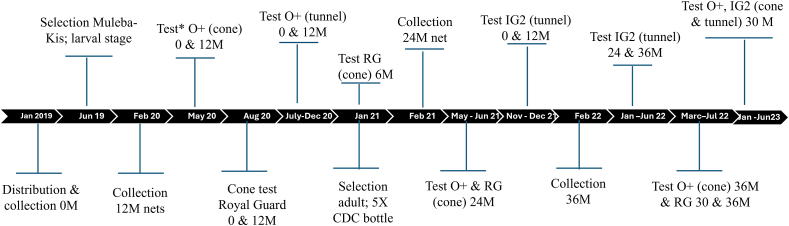
Sampling and testing frame of ITNs collected from the field at different time points. Time of net distribution, collection from the field, and time the test was conducted. At 0, 12 and 24 months, a total of 120 LLINs (30 from each arm) were sampled whereas at 36 months a total of 200 LLINs (50 from each arm) were collected. *Abbreviations*: RG, Royal Guard; O-plus, Olyset Plus; IG2, Interceptor G2; M, month.

In January 2019, over 147,000 ITN (Interceptor, Interceptor G2, Royal Guard, or Olyset Plus) were distributed across the 84 clusters of the randomised controlled trial, in Misungwi District. The description of the Misungwi study area and net sampling is detailed elsewhere (Mosha et al., 2021; Martin et al., 2022). In brief, this study was conducted in 40 of the 84 clusters, 10 clusters per arm. A total of 650 households per arm were enrolled in the study one-month post-distribution. Assuming an average of 3 study nets per household, this would result in 1950 ITNs enrolled. After obtaining participant consent, all study nets in the households were labelled with a unique identification number, and a master list was generated to facilitate net follow-up. A total of 30 nets were sampled at 6, 12, 18, 24, and 30 months, with 50 nets collected at 36 months (Martin et al., 2022). All collected nets were replaced with new nets of the same type. These new nets were excluded from the study; however, the households remained part of the study until no enrolled nets remained. Testing was prioritised for time points 0, 12, 24, and 36 months (Fig. 1). Intermediate time points at 6, 18, and 30 months were tested if the annual time point failed the cone or tunnel test.

The ITNs sampled (Martin et al., 2022) were prepared following the WHO guidelines (WHOPE, 2013). Each net was given a unique identification number based on household and net numbers. For new unwashed nets, 3 adjacent pieces, each measuring 30 × 30 cm, were collected from each side of the net which means 15 pieces per net (WHOPE, 2013). At subsequent time points, the side panel from which the pieces were cut from the bottom (position 1) was excluded due to potential abrasion, leading to a total of 12 net pieces collected. From the 3 adjacent pieces collected in each side, one underwent chemical analysis *via* high-performance liquid chromatography (HPLC) at a Singapore-accredited laboratory, another was tested against susceptible mosquitoes, and the third against resistant mosquitoes. Samples were labelled with household number, net type, net number, net position, time point, and preparation date, then stored in a refrigerator at 4–8 °C before and after testing.

Testing was conducted at the Pan African Malaria Vector Research Consortium (PAMVERC) facility in the National Institute for Medical Research (NIMR), Mwanza, with susceptible *An*. *gambiae* (*s.s*.) Kisumu strain and at the Kilimanjaro Christian Medical University College (KCMUCo) facility in lower Moshi with the resistant *An. gambiae* (*s.s.*) Muleba-Kis strain. The tests were conducted according to the WHO LLIN testing guidelines (WHOPE, 2013).

### 2.3 Characterization of mosquitoes

The study employed the susceptible (Kisumu) and resistant (Muleba-Kis) strains of *An. gambiae* (*s.s.*). The Muleba-Kis colony was established by mating F1 wild male *An. gambiae* mosquitoes with susceptible female *An. gambiae* (*s.s.*), followed by larval selection using pyrethroid (alpha-cypermethrin) insecticide at varying concentrations (Azizi et al., 2021). This strain exhibited both target-site resistance, mainly *kdr*-East L1014S, and metabolic resistance i.e. mixed function oxidases-based resistance, serving as a model to assess the efficacy of the partner AI. The colony was regularly exposed to pyrethroid selection pressure, with monitoring of phenotypic and genotypic resistance to track changes in resistance frequency and intensity.

For the mosquitoes used to test Olyset Plus and Royal Guard dual-ITNs, selection was done once per generation at the larval stage, using 0.08 μg/ml of alpha-cypermethrin. To increase resistance, as the difference in mortality between Interceptor G2 and standard pyrethroid LLIN with the Muleba-Kis selected at 0.08 μg/ml was not large enough, additional selection was done at the adult stage using a 5-fold diagnostic dose of alpha-cypermethrin in Centres for Disease Control (CDC) bottles. Throughout the study, all mosquitoes were maintained in a controlled environment with a temperature range of 25–29 °C and a relative humidity (RH) of 60–95%.

### 2.4 Testing procedures

For the cone test, each 30 cm × 30 cm sample of netting was fixed to a cone frame positioned at an angle of 45° during exposure (Owusu et al., 2016). Mosquitoes (either susceptible or resistant) aged 2–5 days were exposed for 3 min during the daytime and then transferred into paper cups with access to 10% sugar solution. An untreated net and a standard Interceptor net were run in parallel to dual-AI ITNs during each test. The bioassays were carried out at a RH of 75 ± 10% and a temperature of 27 ± 2 °C. Knockdown (60 min) and mortality at 24, 48 and 72 h were reported for all ITNs tested (Table 1). The effect of pyriproxyfen was assessed by blood-feeding mosquitoes before exposure to treatment. Egg development stage determined by ovarian dissection was reported at 72 h for Royal Guard ITNs, positive control (standard Interceptor LLIN) and negative control (untreated net) (Soto et al., 2023).

**Table 1:** Test plan for the active ingredients in study ITNs.

Net type	Active ingredient	Strain	Primary test method	Primary outcomes
Interceptor G2	Chlorfenapyr	Muleba-Kis	Tunnel	72-h mortality and blood-feeding inhibition
	Alpha-cypermethrin	Kisumu	Cone	60-min knockdown and 24-h mortality
Royal Guard	Pyriproxyfen	Muleba-Kis	Cone	Sterility assessed by dissection of ovary after 72 h
	Alpha-cypermethrin	Kisumu	Cone	60-min knockdown and 24-h mortality
Olyset Plus	Piperonyl-butoxide	Muleba-Kis	Cone	60-min knockdown and 24-h mortality
	Permethrin	Kisumu	Cone	60-min knockdown and 24-h mortality

*Note*: Tunnel test was done if the cone test did not meet the WHO threshold with ≥95% knockdown or ≥80% mortality after 24 h, or tunnel tests showed ≥80% mortality or ≥90% inhibition of blood-feeding.

If the mortality rate in the negative control was over 10%, the experiment was repeated. For each ITN sample, between 80 and 100 mosquitoes were exposed. The pyrethroid component was assessed for all the net samples (Royal Guard, Olyset Plus, Interceptor G2) using the susceptible *An. gambiae* Kisumu strain. To assess the partner insecticide, cone tests were conducted for Royal Guard and Olyset Plus, while tunnel tests were used for Interceptor G2 against the Muleba-Kis resistant colony (Martin et al., 2022). Of the three treatments, Interceptor G2 net was the only net tested directly in tunnel tests due to its slow mode of action that needs longer exposure (Black et al., 1994). Otherwise, tunnel tests were carried out on the nets that did not meet the 95% knockdown and/or 80% mortality in the cone bioassays as per the WHO recommendation (WHOPE, 2013). Two replicates on the net piece that yielded mortality closest to the mean mortality observed during the cone test were tested in the tunnel test (Martin et al., 2022). Nine holes (1 cm in diameter) were cut in each net piece. The net was fitted in the tunnel holding frame available for mosquitoes. A guinea pig was restrained in a cage at the shorter part of the tunnel (WHOPE, 2013) as a blood-meal source for mosquitoes. A total of 50 non-blood-fed mosquitoes aged 5–8 days were introduced at the opposite end of the tunnel to the guinea pig (release chamber). The experiment began at 18:00 h and ended at 8:00 h the following morning. Testing was conducted at a RH of 80 ± 20% and a temperature of 27 ± 2 °C. In the morning, the mosquitoes were collected using a mouth aspirator and placed in a separate paper cup per physiological status (i.e. blood-fed, unfed) and dead or alive from each chamber, and supplied with 10% glucose. Blood-feeding rate was recorded in the morning of mosquito collection and mortality was recorded after 24, 48 and 72 h post-exposure. If mortality in control was >10% or blood-feeding <50%, then the test was repeated.

Mosquitoes for Royal Guard were blood-fed on guinea pigs before exposure in cone assays and only successfully blood-fed (fully engorged) mosquitoes were exposed. After exposure, mosquitoes were monitored for 72 h to allow egg maturation. During the normal gonotrophic cycle, after taking a blood meal, mosquito oocytes change in size and shape and finally reach Stage V, which is a distinctive crescent shape (Fowler et al., 2021). The effect of pyriproxyfen on reproductive outcome was assessed by dissecting gravid *An. gambiae* (*s.s.*) after exposure when eggs should normally have been fully matured (Fowler et al., 2021). A mosquito was recorded as fertile if eggs reached stage V and infertile (immature) if mosquito eggs were observed in stages I to IV.

### 2.5 Resistance intensity assays for alpha-cypermethrin and permethrin

To monitor insecticide resistance heterogeneity in the colony of mosquitoes used for cone and tunnel tests, *An. gambiae* (*s.s.*) Muleba-Kis were subjected to different concentrations (1 × , 2 × , and 5 × ) of alpha-cypermethrin and permethrin for 30 min in CDC bottle assays (Additional file S1: Supplementary Tables S1 and S2). If the mortality rate at 5 × was below 97%, further testing at 10 × was conducted. Knockdown observations were documented at 15, 30 and 60 min. Following the 30-min exposure, surviving *An. gambiae* (*s.s.*) from treated and control containers were transferred into paper cups with cotton wool soaked in a 10% glucose solution. The knocked-down or dead *An. gambiae* (*s.s.*) were also separated into cups with the same glucose solution in case of potential revival. Mortality/revival assessments were conducted at 24, 48, and 72 h.

### 2.6 Data analysis

The bioassay data were recorded on standardized forms and double entered into a Microsoft Access file to ensure accuracy. The analysis was done using Stata software version 18. Proportional knockdown (KD) and mortality (24 h) or blood-feeding inhibition (BFI) were presented with their respective 95% confidence intervals (CI) and net pass or fail reported. Since there are no thresholds set up for dual-AI ITN against resistant *Anopheles*, at each time point we compared mortality (24 or 72 h) or sterility (for Royal Guard) between standard pyrethroid LLIN Interceptor and the dual-AI or synergist nets. The comparison in mortality between the dual-AI ITNs or PBO ITN and the standard LLIN was done using multilevel mixed-effects generalized linear models with test number as a random effect and net type as a fixed effect. Odds ratio and *P*-value were reported.

In the resistance assays, mortality was expressed as the proportion of dead mosquitoes across all exposure replicates of the total number of exposed mosquitoes. A parallel calculation was executed to derive the percentage of control mortality. The mortality of 5–20% observed in control assays was adjusted using Abbottʼs formula. Dose-response analysis involved the utilization of a log-probit statistical model to estimate lethal doses (LD_50_ and LD_90_) along with their corresponding 95% confidence intervals.

## 3 Results

### 3.1 Bio-efficacy results against the susceptible strain

**Fig. 2:**
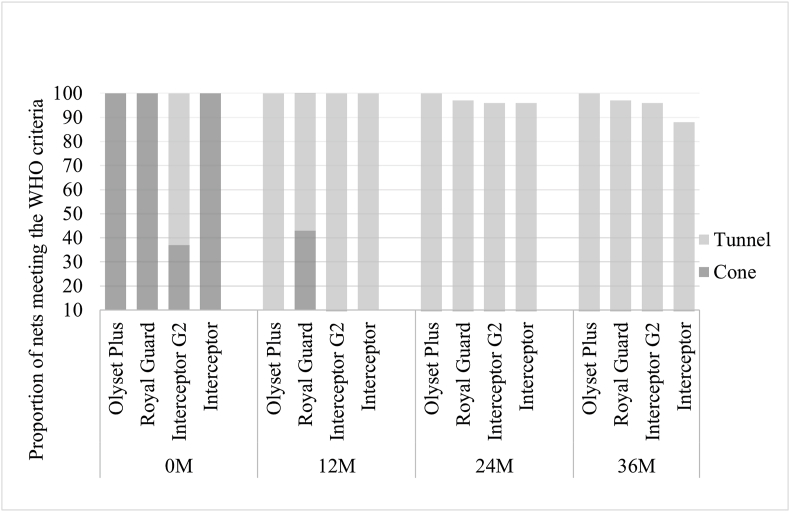
Proportion of nets that passed the WHO threshold 24 h post-exposure in cone and tunnel tests against susceptible *An. gambiae* (*s.s.*) Kisumu strain. *Abbreviation*: M, month.

The proportion of nets that passed the WHO threshold in cone tests against susceptible mosquitoes was 100% for standard LLIN, 37% for Interceptor G2, 100% for Olyset Plus, and 100% for Royal Guard when new (Fig. 2; Additional file S1: Supplementary Table S3). This proportion decreased at each time point, with no net passing at 36 months. In tunnel tests at 36 months, the corresponding percentages were: 88% for Interceptor, 96% for Interceptor G2, 100% for Olyset Plus, and 95% for Royal Guard. Most nets met the bioassay criteria through blood-feeding inhibition (75%, 361/480) in tunnel test rather than mortality (10%, 46/480) (see Additional file S1: Supplementary Tables S3 and S4).

### 3.2 Bio-efficacy results against resistant *An. gambiae* (*s.s.*) Muleba-Kis exposed to Olyset Plus ITNs

**Fig. 3:**
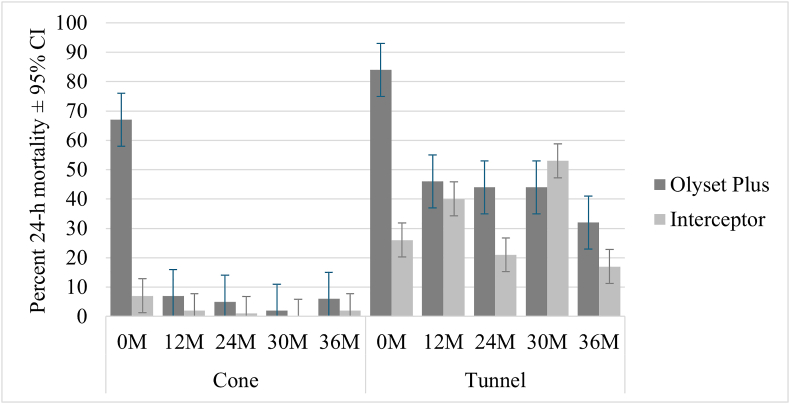
Percent 24-h mortality recorded in Olyset Plus against *An. gambiae* (*s.s.*) resistant strain in cone and tunnel tests. *Abbreviations*: CI, confidence interval; M, month.

When Olyset Plus was new, 60-min knockdown against resistant *An. gambiae* (*s.s*.) Muleba-Kis recorded in the cone assay was 96% compared to 17% observed in standard Interceptor while 24-h mortality was 67% *vs* 7% for Interceptor nets (Fig. 3, Additional file S1: Supplementary Table S5). At the subsequent time points, most of the Olyset Plus nets had to be tested in the tunnel assay. In the tunnel tests, mortality was 84% when new, decreasing to 46% at 12 months, 44% at 24 months, and 33% at 36 months. Mortality was significantly higher in Olyset Plus compared to standard Interceptor nets at 0, 24 and 36 months (Fig. 3). In the tunnel tests, mortality in Interceptor nets varied between 53% and 17% with no trend related to net age. However, the highest mortality was reported at 12 and 30 months, which might explain the lack of difference between Olyset Plus and Interceptor at these time points.

### 3.3 Bio-efficacy results against resistant *An. gambiae* (*s.s.*) Muleba-Kis exposed to Royal Guard ITNs

**Fig. 4:**
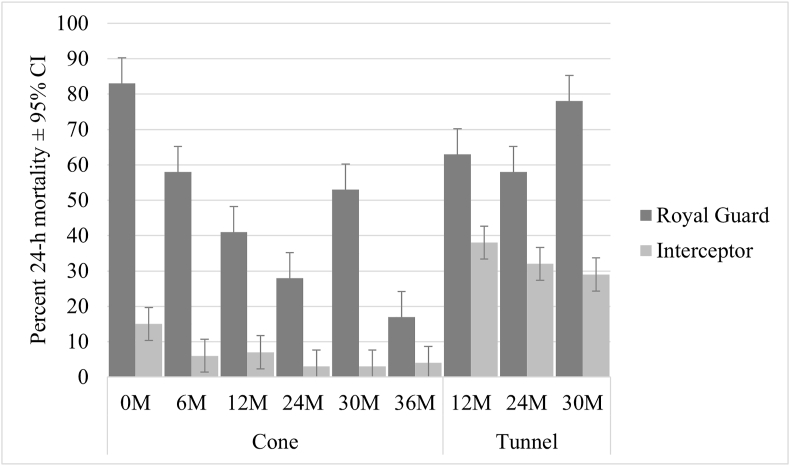
Percent 24-h mortality recorded in Royal Guard against *An. gambiae* (*s.s*.) resistant strain in cone and tunnel tests. *Abbreviations*: CI, confidence interval; M, month.

In both cone and tunnel tests, the 24-h mortality of resistant *An. gambiae* (*s.s*.) was significantly higher after exposure to Royal Guard compared to standard Interceptor at all time points. In the cone tests, mortality was 83% when Royal Guard was new and decreased to 17% after 36 months (Fig. 4, Additional file S1: Supplementary Table S5). Mortality in tunnel tests was generally higher than in cone tests at the same time points. There was a small proportion of mosquitoes surviving (8%) after the 72-h post-exposure period, resulting in a limited number of mosquitoes available for dissection at month zero. The sterility effect in cone tests was 88% with new unwashed Royal Guard nets, 46% at 6 months, and subsequently ranged from 5% to 2%. Given the fact that the sterility effect did not increase with longer exposure in tunnel tests at 24 months (< 5%), tunnel tests were not performed at 36 months.

### 3.4 Bio-efficacy results against resistant *An. gambiae* (s.s.) Muleba-Kis exposed to interceptor G2 ITNs

**Fig. 5:**
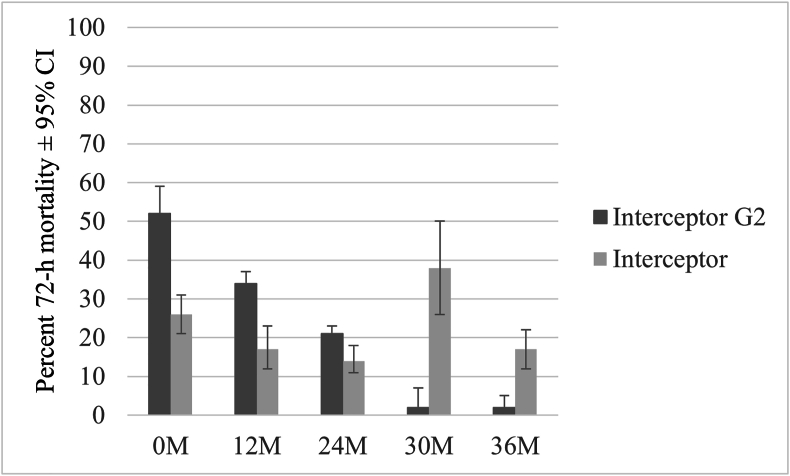
Percent 72-h mortality recorded in Interceptor G2 against *An. gambiae* (*s.s.*) resistant strain in tunnel test. *Abbreviations*: CI, confidence interval; M, month.

Mortality (72 h) in the Muleba-Kis resistant strain exposed to Interceptor G2 ranged from 52% (95% CI: 45–58%) when new to 20% (95% CI: 17–24%) at 36 months. Mortality was significantly higher in Interceptor G2 compared to standard LLIN up to the 24-month time point, with the largest difference observed when the net was new (Fig. 5, Additional file S1: Supplementary Table S5).

### 3.5 Blood-feeding inhibition in the resistant strain Muleba-Kis

**Fig. 6:**
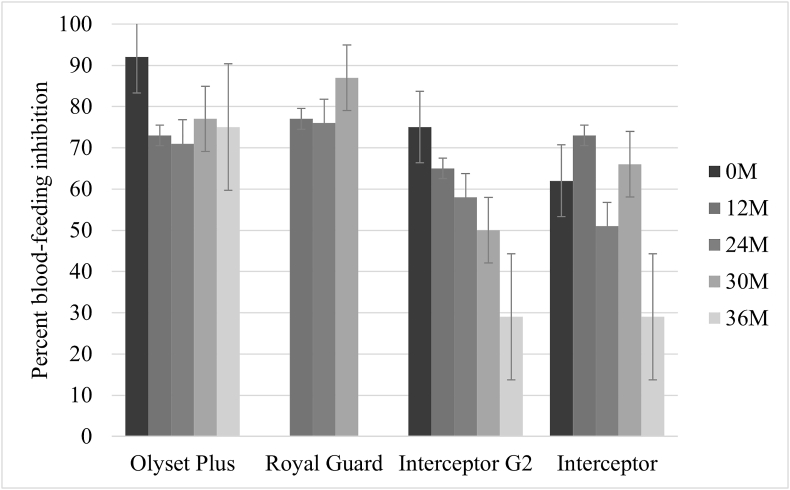
Percent blood-feeding inhibition in the resistant strain induced by each net at different time points. *Abbreviation*: M, month.

The blood-feeding rate in the resistant Muleba-Kis strain was 94% for untreated net exposed in tunnel tests. The highest blood-feeding inhibition (BFI) was observed for Olyset Plus and Royal Guard nets across all time points. BFI varied between 71% and 92% for Olyset Plus and between 76% and 87% for Royal Guard. Similar to mortality, BFI was higher at 0, 24 and 36 months for Olyset Plus compared to standard LLIN. For Royal Guard, when tunnel tests were performed at 12, 24, and 30 months, BFI was also higher than for standard LLIN, with the largest difference observed at 24 months. For Interceptor G2, BFI was 75% when new and decreased to 29% after 36 months, remaining similar to Interceptor at all time points (Fig. 6).

### 3.6 Insecticide resistance intensity

In the first year, the concentration (LD_50_) of permethrin needed to kill 50% of Muleba-Kis strain (selected at the larval stage using 0.08 μg/ml of alpha-cypermethrin) was 31.1 μg/ml (95% CI: 22.4–39.7). By the third year, LD_50_ was 96.2 μg/ml (95% CI: 86.9–105.9), indicating an increase in insecticide resistance in the colony (see Additional file S1: Supplementary Table S6). For alpha-cypermethrin, the LD_50_ was 12.4 μg/ml (95% CI: 9.7–15.1) during the first year, decreasing to 5.9 μg/ml (95% CI: 4.9–6.9) in the third year and 5.6 μg/ml (95% CI: 3.6–7.5) in the fourth year. Mosquitoes from the first year were used to test Olyset Plus and Royal Guard at 0, 12 and 24 months of use, while mosquitoes from the third and fourth years were used for testing nets at 30 and 36 months of use. The decrease in alpha-cypermethrin resistance over time might explain the increase in mosquito mortality after exposure to Interceptor. Combined with the rise in permethrin resistance, this could explain why the difference in mortality between Interceptor and Olyset Plus was less pronounced in Years 3 and 4.

Mosquitoes selected at the adult stage with the 5x diagnostic dose of alpha-cypermethrin showed no mortality after exposure to a 1x diagnostic dose (12.5 μg/ml) of alpha-cypermethrin during the first year of testing. They were not further tested for resistance in Years 3 and 4, so variation in resistance during these years is not known.

## 4 Discussion

The bio-efficacy of Interceptor G2, Royal Guard, and Olyset Plus was compared to a standard pyrethroid LLINs (Interceptor) at each time point over three years under community use conditions. Overall, the killing effects of these nets were superior to the standard pyrethroid LLIN against resistant vectors over time. Olyset Plus demonstrated higher mortality than the standard LLIN when new, and at 24 and 36 months in tunnel tests. Royal Guard also consistently showed higher mortality and delayed mortality (72 h) but had limited sterility effects only for the first six months compared to the standard LLIN. Interceptor G2 was superior to Interceptor for up to two years.

More than 80% of the tested ITNs met the WHO bio-efficacy criteria in cone and tunnel tests against the susceptible colony strain. They achieved the WHO criteria primarily through blood-feeding inhibition in tunnel tests against the susceptible *An*. *gambiae* (*s.s*.) rather than mortality or knockdown. In this study, the bio-efficacy of dual-AI ITNs against susceptible mosquitoes was sustained for three years, meeting WHO expectations.

In the present study, the stronger and longer-lasting impact observed for Royal Guard on resistant *An*. *gambiae* (*s.s*.) was on mortality rather than sterility. Royal Guard induced mortality was significantly higher than that of the standard Interceptor net over time. Interestingly, in a study conducted in Benin, an ITN with only pyriproxyfen induced some mortality (ranging from 47% when new to 28% after 20 washes) (Ngufor et al., 2020). The impact of pyriproxyfen on mortality has also been reported elsewhere (Grisales et al., 2021). This may explain the superior killing effect observed in the present study if there was some additive effect of the pyrethroid and pyriproxyfen. However, the findings of the present study contradict laboratory and experimental hut data from other studies (Ngufor et al., 2020; Tchouakui et al., 2023) which did not report a difference in mortality between Royal Guard and standard LLINs. In Benin, mortality in cone tests was very high for both nets (over 80%). In EHTs in Benin and in the study conducted in Cameroon (Tchouakui et al., 2023), mortality was around 20% for both nets (depending on species and study area). Differences between the studies could be attributed to insecticide resistance, and the fact that the reference net (Interceptor) in the present study uses a different treatment technology than Royal Guard, which could lead to differences in insecticide surface concentration and thus killing effect. Another notable difference with other studies was the impact of Royal Guard on sterility. In the present study, a significant sterility effect (88%) was observed in surviving mosquitoes 72 h after exposition to Royal Guard in cone assays, and this effect was reduced by half after six months. In Benin, a reduction in offspring was observed with new and after 20 washes Royal Guard compared to the control net (Ngufor et al., 2020). Another study with a different brand reported a longer-lasting sterility effect (Ngufor et al., 2016). These discrepancies could be explained by the washing methods of the nets, as nets in the laboratory are not exposed to the abrasive washing conditions typically found in community settings. This study highlighted that laboratory wash resistance does not reflect the performance of nets after 36 months in operational settings due to differences in washing and drying techniques. Additionally, during data collection for this study, no standard guidelines existed for assessing the effects of pyriproxyfen on egg development. As a result, eggs at stages I to IV were classified and reported as immature (Lissenden et al., 2021), while only stage V was reported as fertile. Recently, a WHO implementation guideline (WHO, 2023c) was updated to classify both stages IV and V as fertile, which was not reflected in this study. This may explain the lower sterility effect reported in this study compared to the standard assessment of fertility through oviposition.

Olyset Plus outperformed the control (standard Interceptor net) when new, and then again at 24 and 36 months of use. In another community durability study conducted in Uganda, the superior bio-efficacy of field Olyset Plus nets compared to standard LLIN was reported for up to two years (Mechan et al., 2022). Similarly, a study in Kenya, assessing the bio-efficacy and durability of PBO-based nets found that Olyset Plus nets performed better or close to WHO critical threshold for about two years in the field (Gichuki et al., 2021).

Interceptor G2 demonstrated superiority over Interceptor net up to 24 months. After 24 and 36 months, no statistical difference was observed between Interceptor G2 and Interceptor. At 30 months, Interceptor seemed to outperform Interceptor G2. This testing was conducted at the end of the study, after all other time points had been completed. The observed results may be due to a reduction in alpha-cypermethrin resistance in the mosquito colony strain, leading to higher mortality in mosquitoes exposed to the standard LLIN Interceptor. Interceptor G2 induced lower mortality compared to other studies (NʼGuessan et al., 2016; Bayili et al., 2017). However, the observed mortality was comparable to the 50–60% mortality reported in the associated EHT study (Tungu et al., 2021) when the nets were new and remained relatively similar at other time points. Studies conducted in Cameroon (Black et al., 1994), Tanzania (Tungu et al., 2021), and Benin (NʼGuessan et al., 2016) using washed nets reported mortality around 80% when new, with Interceptor G2 showing superior bio-efficacy even after 20 washes. Differences in mortality among studies could be due to varying levels of resistance intensity and the colony species strain used in these countries. Additionally, 20 washes of nets do not reflect 36 months of net use in a community setting due to a high depletion of insecticide during washing in the community. In the present study, it was particularly challenging to select a colony of *An. gambiae* (*s.s.*) that was resistant enough to see a difference in mortality between Interceptor G2 and the reference standard LLIN, and secondly, to maintain the resistance intensity constant over the three years of testing. Standard operating procedures (SOPs) (Lissenden et al., 2021; Lees et al., 2022) have been established to guide testing, but further considerations are needed to reduce variation in mosquito strains used in the testing. One approach could be shortening the testing duration and testing all time points of the same net type side by side. Lastly, a key difference from other studies is that the nets used in the present study were sourced from a much larger batch than those in other studies. Typically, nets used for wash resistance assays are provided by manufacturers in small quantities specifically for the research. In contrast, our study used nets manufactured in larger quantities, which might result in greater variability between batches.

In the present laboratory study, Royal Guard and Olyset Plus outperformed the standard LLIN in terms of entomological bio-efficacy in tunnel tests, maintaining efficacy the longest, while the superior efficacy of Interceptor G2 ended after two years. Interestingly, in terms of impact on malaria in the associated community cRCT, Interceptor G2 was the most effective in epidemiological and entomological outcomes for three years. Olyset Plus outperformed the standard LLIN for only one year, and Royal Guard did not show a superior effect in the cRCT (Mosha et al., 2022).

Ideally, supplementary evidence on the entomological mode of action of the partner active ingredients is needed to provide greater insight into the class of net’s capacity to control malaria transmission. For Interceptor G2, there may be growing evidence that, in addition to killing vectors, the chlorfenapyr component also affects *Plasmodium* inside the vector (Kweyamba et al., 2023; Nʼ;Guessan et al., 2023). For Olyset Plus, there may be a residual repellence effect at low concentrations, evident in the tunnel tests but not in the community where PBO net usage was low due to quality issues (Protopopoff et al., 2023). With Royal Guard, the net is designed to control the F1 larval population, which may also be regulated by density-dependent competition for planktonic food resources, potentially undermining control.

Malaria transmission models that rely only on experimental hut trials may not be sufficient to parameterise the full transmission model and may need supplementary entomological laboratory outcomes to provide insight into what information may be missing. There is a continuing need for cRCT trials to provide definitive epidemiological evidence on the level of effect and the mode of entomological action.

## 5 Conclusions

Although all nets tested met the WHO criteria for up to three years against a susceptible strain of *Anopheles*, this was mainly through blood-feeding inhibition in tunnel tests rather than mortality in cone tests. Overall, next-generation ITNs demonstrated a higher effect on mortality compared to standard LLINs, the superior efficacy did not last for three years and varied according to net brands. This suggests that the effect of the second ingredient of the dual-AI and piperonyl butoxide (PBO) were not sustained. To address this issue, further development is required to enhance the long-lasting bio-efficacy of the second ingredient of the dual-AI.

## Funding

This study was funded under the joint global health trial from the 10.13039/501100000276Department of Health and Social Care, the Department for International Development, the Medical Research Council and 10.13039/100010269Wellcome Trust (#MR/R006040/1), and the 10.13039/100000865Bill and Melinda Gates Foundation through Innovative Vector Control Consortium (IVCC).

## Ethical approval

This study was nested in a larger cRCT conducted in Misungwi. The cRCT received ethical approval from Kilimanjaro Christian Medical Collage, the National Institute for Medical Research (NIMR/HQ/R.8a/Vol.IX/2743), and the London School of Hygiene and Tropical Medicine (Ref: 16524) on April 16, 2020. Informed Consent: consent was obtained from the head of household or a person above 18 years before net collection. For those who agreed to be part of the study, a fingerprint/signature was taken, and the net was collected by a field worker and replaced by a new net.

## Data availability

The data supporting the conclusions of this article are included within the article and its supplementary files. The raw data will be available in the LSHTM repository dataset after completing an ongoing secondary analysis.

## CRediT authorship contribution statement

**Jackline L. Martin:** Conceptualization, Methodology, Formal analysis, Investigation, Writing – original draft, Writing – review & editing, Visualization, Supervision. **Louisa A. Messenger:** Conceptualization, Methodology, Formal analysis, Investigation, Writing – original draft, Writing – review & editing, Visualization, Supervision. **Edmund Bernard:** Investigation, Writing – review & editing. **Monica Kisamo:** Investigation, Writing – review & editing. **Patric Hape:** Resources, Investigation, Writing – review & editing. **Osca Sizya:** Investigation, Writing – review & editing. **Emmanuel Festo:** Investigation, Writing – review & editing. **Wambura Matiku:** Investigation, Writing – review & editing. **Victoria Marcel:** Resources, Investigation, Writing – review & editing. **Elizabeth Malya:** Investigation, Software, Data curation, Writing – review & editing. **Tatu Aziz:** Investigation, Writing – review & editing. **Nancy S. Matowo:** Investigation, Supervision, Writing – review & editing. **Jacklin F. Mosha:** Project administration, Investigation, Supervision, Writing – review & editing. **Franklin W. Mosha:** Project administration, Investigation, Supervision, Writing – review & editing. **Mark Rowland:** Funding acquisition, Conceptualization, Methodology, Investigation, Writing – original draft, Writing – review & editing. **Alphaxard Manjurano:** Project administration, Investigation, Supervision, Writing – review & editing. **Natacha Protopopoff:** Conceptualization, Methodology, Formal analysis, Investigation, Writing – original draft, Writing – review & editing, Visualization, Supervision.

## Declaration of competing interests

The authors declare that they have no known competing financial interests or personal relationships that could have appeared to influence the work reported in this paper.

## Data Availability

The data supporting the conclusions of this article are included within the article and its supplementary files. The raw data will be available in the LSHTM repository dataset after completing an ongoing secondary analysis.
