# Supplementary material for: Evaluation of bio-efficacy of field-aged novel long-lasting insecticidal nets (PBO, chlorfenapyr or pyriproxyfen combined with pyrethroid) against Anopheles gambiae (s.s.) in Tanzania
Source: Curr Res Parasitol Vector Borne Dis. 2024 Sep 23;6:100216. doi: 10.1016/j.crpvbd.2024.100216 (PMC11470491; doi:10.1016/j.crpvbd.2024.100216)
Supplement: Multimedia component 1 [file mmc1.pdf]

Additional file S1

Supplementary Table S1. CDC bottle assay: concentration used and number of *Anopheles* exposed to α-cypermethrin.

Replicates	Insecticide	Concentration	No. of *Anopheles* per bottle
Bottle 1 (1×)	α-cypermethrin	12.5 µg/ml	15–20
Bottle 2 (2×)	α-cypermethrin	25 µg/ml	15–20
Bottle 3 (5×)	α-cypermethrin	62.5 µg/ml	15–20
Bottle 4 (10×)	α-cypermethrin	125 µg/ml	15–20
Bottle 5	Control (Acetone)	1ml	15–20

Supplementary Table S2. CDC bottle assay: concentration used and number of *Anopheles* exposed to permethrin.

Replicates	Insecticide	Concentration	No. of *Anopheles* per bottle
Bottle 1 (1×)	permethrin	21.5 µg/ml	15–20
Bottle 2 (2×)	permethrin	43 µg/ml	15–20
Bottle 3 (5×)	permethrin	107.5 µg/ml	15–20
Bottle 4 (10×)	permethrin	215 µg/ml	15–20
Bottle 5	Control (Acetone)	1ml	15–20

Supplementary Table S3. Cone and tunnel test results conducted for each type of net (Interceptor, Interceptor G2, Royal Guard, and Olyset Plus) against the susceptible *An. gambiae* (*s.s.*) strain at 0, 12, 24, and 36 months post-distribution.

Bio-efficacy of Alphacypermethrin: Cone test with susceptible kisumu strain																
Treatments	Interceptor				Interceptor G2				Royal Guard				Olyset Plus			
Net age	0	12	24	36	0	12	24	36	0	12	24	36	0	12	24	36
N ITNs tested	30	30	30	50	30	30	30	50	30	30	30	50	30	30	30	50
N pieces tested	150	120	120	200	150	120	120	200	150	120	120	200	150	120	120	200
N exposed	2868	2347	2349	7242	2527	2350	2356	4021	2968	2365	2343	3970	2841	2302	2374	3896
N KD	2834	1589	911	1554	2287	1011	645	532	2956	2004	1107	2012	2841	1612	936	587
N dead	2749	1224	596	873	971	713	490	262	2959	1729	614	1242	2817	1275	669	494
% KD	99 (98 - 99)	68 (63 - 72)	39 (34 - 43)	21 (20 - 23)	91 (88 - 93)	43 (37 - 49)	27 (23 - 31)	13 (10 - 17)	100 (99 - 100)	85 (80 - 89)	47 (42 - 52)	51 (45 - 57)	100	70 (65 - 75)	39 (34 - 44)	15 (11 - 19)
% dead	96 (94 - 98)	52 (46 - 58)	25 (22 - 29)	12 (11 - 13)	38 (33 - 44)	30 (25 - 36)	21 (19 - 23)	6 (4 - 8)	100 (99 - 100)	73 (66 - 80)	26 (22 - 31)	31 (25 - 37)	99 (98 -100)	55 (50 - 61)	28 (25 - 31)	13 (9 - 16)
N passed cone	30	3	0	0	11	0	0	0	30	13	0	1	30	3	0	0
% passage	100	10	0	0	37	0	0	0	100	10	0	3	100	10	0	0
Tunnel test with failled nets (<80% mort or KD<95%) from cone bioassay																
Net age	0	12	24	36	0	12	24	36	0	12	24	36	0	12	24	36
N ITNs tested	15	27	30	50	19	30	30	50	NA	27	30	49	NA	27	30	50
N pieces tested	15	27	30	93	19	30	30	50	NA	27	30	49	NA	27	30	50
N exposed	939	2398	2608	4496	2802	2763	2238	4817	NA	1561	2755	3704	NA	2495	2809	4649
N dead	640	1770	1560	2963	1924	2152	1306	3063	NA	1175	1563	2491	NA	1908	1809	3194
N blood-fed	10	28	37	71	40	22	20	64	NA	9	28	37	NA	42	7	18
% dead	68 (63 - 73)	74 (71 - 77)	60 (55 - 64)	66 (64 - 68)	69 (66 - 72)	78 (74 - 82)	58 (55 - 62)	64 (62 - 66)	NA	75 (72 - 78)	57 (52 - 62)	67 (65 - 69)	NA	76 (75 - 78)	64 (60 - 69)	69 (66 - 71)
% blood-fed	1 (0 - 2)	1 (0 - 2)	1 (0 - 2)	2 (0 - 2)	1 (0 - 2)	1 (0 - 1)	0 (0 - 2)	1 (0 - 2)	NA	0 (0 - 1)	1 (0 - 2)	0 (0 - 2)	NA	2 (0 - 3)	0	0
%BFI	NA	98.1	97.3	96.9	97.3	98.1	98.1	97.5	NA	98.1	98	98	NA	96.9	100	100
N passed Tunnel	NA	27	30	50	19	30	30	50	NA	27	30	49	NA	27	30	50
% passage	NA	100	100	100	100	100	100	100	NA	100	100	100	NA	95	100	100

Note: All mortality data are reported at 24 hours post-exposure. Only nets that failed the WHO criteria in cone bioassays were further tested in tunnel assays.

Supplementary Table S4. Results from multilevel mixed-effects generalized linear models comparing mortality of **susceptible** *An. gambiae* (*s.s.*) between each dual-AI/PBO net and standard LLINs of the same age, measured in cone and tunnel test.

	Cone test					Tunnel test			
	Net age	24-h % Mortality	Odds ratio	95% CI	P-value	% Mortality	Odds ratio	95% CI	P-value
Olyset Plus *vs* Interceptor, 24-h mortality	0M	99% *vs* 91%	5.22	2.9–9.1	<0.001	–	–	–	–
	12M	55% *vs* 48%	1.24	1.1–1.4	0.011	76% *vs* 75%	1.08	0.4–0.9	0.384
	24M	28% *vs* 24%	1.21	0.9–1.5	0.068	64% *vs* 64%	0.99	0.8–1.2	0.872
	36M	13% *vs* 10%	1.25	0.9–1.6	0.064	68% *vs* 66%	1.09	0.2–0.9	0.171
Royal Guard *vs* Interceptor, 24-h mortality	0M	100% *vs* 99%	4.09	1.7–9.7	0.002	–	–	–	–
	12M	73% *vs* 57%	2.09	1.7–2.5	<0.000	75% *vs* 70%	1.28	1.0–1.6	0.023
	24M	26% *vs* 29%	0.82	0.7–0.9	0.035	57% *vs* 58%	0.96	0.8–1.1	0.628
	36M	31% *vs* 10%	4.69	3.7–5.9	<0.000	67% *vs* 63%	1.14	1.0–1.3	0.044
Interceptor G2 *vs* Interceptor, 72-h mortality	0M	59% *vs* 97%	0.03	0.0–0.1	<0.000	92% *vs* 91%	1.02	0.9–1.2	0.767
	12M	49% *vs* 75%	0.28	0.2–0.4	<0.000	91% *vs* 89%	1.22	1.0–1.5	0.032
	24M	43% *vs* 48%	0.83	0.7–0.9	0.023	73% *vs* 72%	1.09	0.9–1.3	0.322
	36M	18% *vs* 22%	0.8	0.7–0.9	0.012	72% *vs* 73%	0.88	0.8–0.9	0.039

Supplementary Table S5. Results from multilevel mixed-effects generalized linear models comparing mortality of **resistant** *An. gambiae* (*s.s.*) between each dual-AI/PBO net and standard LLINs of the same age, measured in cone and tunnel test.

Cone test						Tunnel test			
	Net age	24-h % Mortality	Odds ratio	95% CI	P-value	% Mortality	Odds ratio	95% CI	P-value
Olyset Plus *vs* Interceptor, 24-h mortality	0M	67% *vs* 7%	35.4	10.9–115.0	<0.001	84% *vs* 26%	15.9	10.8–23.4	<0.001
	12M	7% *vs* 2%	2.2	0.4–12.6	0.375	46% *vs* 40%	1.3	0.9–1.7	0.084
	24M	5% *vs* 1%	0.3	0–14.1	0.546	44% *vs* 21%	3.1	2.3–4.2	<0.001
	30M	2% *vs* 0%	1527981	210996.1	<0.001	44% *vs* 53%	0.7	0.5–0.8	0.001
	36M	6% *vs* 2%	3.5	0.8–16.4	0.109	33% *vs* 17%	2.8	2.2–3.3	<0.001
Royal Guard *vs* Interceptor, 24-h mortality	0M	83% *vs* 15%	215.6	26.5–1753.4	<0.001	–	–	–	–
	06M	58% *vs* 6%	44.3	8.7–225.5	<0.001	–	–	–	–
	12M	41% *vs* 7%	19.1	4.9–73.9	<0.001	63% *vs* 38%	3.4	2.4–4.9	<0.001
	24M	28% *vs* 3%	14.7	3.1–70.9	0.001	58% *vs* 32%	3.3	2.6–4.1	<0.001
	30M	53% *vs* 3%	154.8	7.6–3138.5	0.001	78% *vs* 29%	8.5	4.7–15.4	<0.001
	36M	17% *vs* 4%	3.6	0.7–19.1	0.138				
Interceptor G2 *vs* Interceptor, 72-h mortality	0M					52% *vs* 26%	3.0	2.3–4.0	<0.001
	12M					34% *vs* 17%	2.6	1.9–3.5	<0.001
	24M					21% *vs* 14%	1.6	1.2–2.1	0.001
	30M					20% *vs* 38%	0.4	0.3–0.5	<0.001
	36M					20% *vs* 17%	1.2	0.9–1.4	0.14

Supplementary Table S6. Percentage mortality induced by pyrethroid (permethrin and alpha-cypermethrin) against **resistant** *An.gambiae* (*s.s.*) strain in CDC bottle assay.

		1×		2×		5×		10×	
		24 h	72 h	24 h	72 h	24 h	72 h	24 h	72 h
Year 1	Permethrin	43%	68%	64%	86%	60%	74%	86%	91%
	Alpha-cypermethrin	58%	71%	64%	80%	85%	97%	94%	97%
Year 3	Permethrin	14%	18%	18%	29%	49%	59%	83%	90%
	Alpha-cypermethrin	66%	69%	81%	83%	87%	89%	96%	97%
